# Körperkoordinationstest Für Kinder (KTK) for Brazilian Children and Adolescents: Factor Analysis, Invariance and Factor Score

**DOI:** 10.3389/fpsyg.2019.02524

**Published:** 2019-11-19

**Authors:** João Paulo Abreu Moreira, Mariana Calábria Lopes, Marcio Vidigal Miranda-Júnior, Nadia Cristina Valentini, Guilherme Menezes Lage, Maicon Rodrigues Albuquerque

**Affiliations:** ^1^Graduate Program in Physical Education, Universidade Federal de Viçosa, Viçosa, Brazil; ^2^Department of Physical Education, Universidade Federal de Viçosa, Viçosa, Brazil; ^3^Graduate Program in Sports Sciences, Universidade Federal de Minas Gerais, Belo Horizonte, Brazil; ^4^Graduate Program in Human Movement Science, Escola de Educação Física, Fisioterapia e Dança, Universidade Federal do Rio Grande do Sul, Porto Alegre, Brazil; ^5^Department of Physical Education, Universidade Federal de Minas Gerais, Belo Horizonte, Brazil; ^6^Department of Sports, Universidade Federal de Minas Gerais, Belo Horizonte, Brazil

**Keywords:** motor competence, Körperkoordinationstest Für Kinder, validity, factor score, invariance

## Abstract

The decrease in children motor competence, with a consequent reduction in the levels of physical activities and fitness, impacting health negatively, has affected children across countries. In addition to consistent intervention strategies, it is necessary to use appropriate instruments. The Körperkoordinationstest Für Kinder (KTK) is a reliable and low-cost motor coordination (MC) test used in several countries but lacking psychometric evidence in the Brazilian population. The present study investigates the factor structure of KTK in a Brazilian sample; and, compared four possibilities of calculating the factorial score of the test, precisely the sum of the scores, sum of the standard scores, weighted method, and the refined method. The participants of the study consisted of 565 volunteers (49.9% boys), from 5 to 10 (7.93 ± 1.51) years of age, with a body mass index (BMI) means of 17.04 (±2.81). The results showed that the KTK factor structure was adequate to the model for the total sample, by sex, and by age groups. However, the results did not confirm the invariance between sexes and age groups. Besides, our result showed that the sum of the raw scores of the subtests could be used as the factor score method in KTK. In the end, we conclude that the KTK is a valid test to measure the MC of Brazilian children and adolescents, with features that qualify it as a useful instrument both for research and for the practice.

## Introduction

The engagement of children and young people in physical activity has been decreasing in many countries (Dollman et al., 2005), and a part of this population has adopted a predominantly sedentary lifestyle (Photiou et al., 2008). Consequently, there has been a considerable increase in the number of young people who are overweight and have low physical fitness in addition to the rise in the incidence of diseases associated with physical inactivity, such as obesity (Booth et al., 2012) among children and adolescents.

The increase of physical activity levels through the development of children’s motor competence (Stodden et al., 2008) is among the various strategies used. Studies have pointed to positive associations between these two variables, showing that children with high levels of motor competence tend to have a higher engagement in physical activities (Wrotniak et al., 2006; Haga et al., 2008; Lubans et al., 2010). We have to point out that the expression motor competence, according to Cattuzzo et al. (2016), used in a global perspective, contemplates all forms of tasks directed to objectives that involve coordination and control of the human body. Thus, it is essential to stimulate the development of motor competence since childhood (Hoeboer et al., 2016). However, for this to occur in addition to consistent and coherent work, the use of systematic assessment is important to measure the progress of children over the levels of motor competence (Fransen et al., 2014).

Among the reliable measurements to assess motor competence, the Körperkoordinationstest Für Kinder (KTK) is one of the most commonly used (e.g., Bardid et al., 2015; Cattuzzo et al., 2016; Rudd et al., 2016) in children and adolescents. KTK was developed in Germany to test children and adolescents, ranging from 5 to 14 years of age (Kiphard and Schilling, 1974). It is considered a relatively simple test, easy to perform, with objective measures, and low operational cost (Cools et al., 2009), which are characteristics that may favor the expansion of its use for both research purposes and the daily activities of Physical Education teachers and sports coaches. The test encompasses components of motor coordination (MC) and consists of four tasks: (1) walking backward along a balance beam of decreasing width: 6.0, 4.5, and 3.0 cm (WB); (2) two-legged jumping from side to side for 15s (JS); (3) moving sideways on wooden boards for 20 s (MS); and (4) hopping for height (HH), which consists of one-legged hopping over a foam obstacle with increasing height in consecutive steps of 5 cm. The scores obtained in each sub-test are compared to the original normative data and transformed into the motor quotients for each task. The sum of the four standardized item scores obtained results in the overall motor quotient (MQ) of the KTK (Kiphard and Schilling, 1974).

To the best of our knowledge, the factorial structure of the KTK was not tested in Brazilian children and adolescents. Thus, considering the importance of the use of KTK as a motor assessment tool, it seems relevant to test its factorial structure based on data obtained from evaluations performed with Brazilian children and adolescents. Moreover, another aspect that stands out is the fact that the original normative values of KTK were established more than 40 years ago in Germany, taking into consideration economic, social and cultural contexts (Robinson et al., 2015), which are very different from the Brazilian ones.

Another point that deserves investigation is related to the result provided by the KTK, the MQ, which can also be treated as a factor score (DiStefano et al., 2009). According to DiStefano et al. (2009), factorial scores are used in the effort to summarize the results obtained in the various items of an instrument in one or more factors. In the KTK, the MQ summarizes in a single value the results obtained in the four subtests. To summarize a MC in a single value, four different calculation methods can be used with specific strength and limitations. Specifically, related to the sum of the raw scores of the subtests (subtest’s method), when this procedure is adopted, the skills with higher values will weigh more for the calculation of the MC. For example, the maximum score that can be achieved in item WB is 72 points, while in JS, the maximum value predicted for girls is 110 points, that is, different tasks, measured in different units. Regarding the sum of the standard scores, another important conceptual problem remains. The sum of the scores assumes that the subtests have the same importance to the calculation of the MC, which may not be real by the results of the psychometric properties (using Confirmatory Factor Analysis) of the KTK. Thus, other methods could be more indicated to use to calculate the factor score; more specifically using the “Weighted Method” (For more details, see Albuquerque et al., 2017) and the “Refined Method” (For more details, see DiStefano et al., 2009). These procedures may be more robust methods contrasted with the sum of scores and sum of the standard scores.

Therefore, the present study investigates the factor structure of the KTK for a Brazilian sample; and, compared four possibilities of calculating the factorial score of the test, precisely the sum of the scores, sum of the standard scores, weighted method, and the refined method.

## Materials and Methods

### Participants

The participants of the study consisted of 565 volunteers, from 5 to 10 years of age, with age mean of 7.93 (±1.51) years, and a body mass index (BMI) means of 17.04 (±2.81). Of these, 282 (49.9%) were boys, and 283 (50.1%) were girls, all of whom are regularly enrolled in Brazilian public and private schools in Minas Gerais (one state of Brazil) and attending classes from the 1st to 5th grade of elementary school.

The ethical committee of the Institutional Review Board of the Universidade Federal de Viçosa approved the study (26874614.1.0000.5153). Caregivers, those legally responsible for the children were informed about the goals and relevance of the research, as well as the procedures that would be adopted. Besides, they signed the consent authorization for the participation of children.

### Motor Coordination Assessment

The MC of the participants was assessed through the application of KTK - *Körperkoordinationstest Für Kinder*, developed by Kiphard and Schilling (1974). The test involves components of MC, such as balance, rhythm, strength, laterality, speed, and agility (Scordella et al., 2015). The test consists of four tasks: (1) walking backward (WB) along a balance beam with a decreasing width, from 6.0 cm to 4.5 cm, to 3.0 cm; (2) two-legged jumping from side to side for 15 s. (JS); (3) moving sideways on wooden boards for 20 s (MS); and (4) hopping for height (HH), with one-legged, over a foam obstacle with increasing height in consecutive steps of 5 cm (Rudd et al., 2016).

### Procedure

Participants’ full name, sex, and date of birth were obtained using a questionaire. The KTK test was conducted at the participants’ schools. The first task was WB, followed by sub-tests JS, MS, and HH, following all guidelines established by the authors (Kiphard and Schilling, 1974). Assessors, who were responsible for the assessments, underwent training sessions both in groups (two times) and individually (more than four times). The scores of a total of 50 (≅10) participants were used to conduct the raters’ agreement for all subtests. Raters’ reliability was conducted with Cohen’s kappa test, which indicated an agreement above 80% in all cases.

### Statistical Analysis

For the construct validity of the KTK, a confirmatory factorial analysis (CFA) was conducted. Maximum likelihood (ML) estimator was used since it was recommended as an alternative when data are continuous. For the fittest of the proposed model, we assessed the indices of χ^2^ (Chi-square); CFI (comparative fit index); TLI (Tucker-Lewis index); RMSEA (root mean square error of approximation); and SRMR (standardized root mean square residual) following the recommended literature (Hu and Bentler, 1999; Brown, 2006). Recognized values were adopted as criteria for a satisfactory model fit for the data: CFI and TLI less than 0.9; RMSEA with a value close to or less than 0.06; SRMR value close to or less than 0.08 (Hu and Bentler, 1999; Brown, 2006). Also, Cronbach’s Alpha was used to measure Reliability.

A multigroup confirmatory factor analysis (MGCFA), using the ML estimation procedure, was used to test the assumption of the KTK invariance across sex and age groups. Factorial invariance testing followed a series of hierarchical steps, each comprising consecutive constraints across sex. An initial confirmatory analysis tested the proposed model in each sex separately. In addition, it was tested whether the same parameters existed for both sexes (configural invariance). Moreover, factor loadings (metric invariance), item intercepts (scalar invariance), and residual variances (strict invariance) were investigated (Hirschfeld and von Brachel, 2014). As recommended by many authors (e.g., Brown, 2006; Kline, 2011), the model fit was evaluated using (a) χ^2^ goodness-of-fit; (b) root mean square error of approximation (RMSEA; with values lower than.08 being indicative of acceptable fit to the data); and (c) comparative fit index (CFI; with values greater than 0.90). A change of lower than 0.01 in CFI between configural and metric invariance models, in addition to a change of lower than 0.02 in RMSEA, indicated non-invariance, while a change of lower than 0.01 and 0.02 for CFI and RMSEA, respectively, would confute scalar or strict invariance (Vandenberg and Lance, 2000).

The sum of the scores of all samples and separated by sexes were calculated by the sum of raw scores of the subtests (Equation 1). Where WB, SJ, MS, and HH represent Walking Backward, Jumping Sideways, Moving Sideways, Hopping raw data, respectively.

(1) MQ=s⁢u⁢mWB+SJ+MS+HH

Regarding the Sum of the Standard Scores, to use only positive numbers, we chose to transform each raw value in a “min-max scaling” (Equation 2), which transforms the data such that the values are within a specific range [0 to 1]. In which, x’ is the normalized value; x is the raw data, xmin is the minimum value found in the sample, and xmax is the maximum value of the sample. This procedure was conducted for each subtest separately. Subsequently, the transformed data values of each subtest were summed to calculate the MQ_standard_ (Equation 3).

(2) $$x^{{}^{\prime }}=\frac{x-\mathrm{xmin}}{\mathrm{xmax}-\mathrm{xmin}}$$

(3) MQ=standardWBx′+SJx′+MSx′+HHx′

Weighted method (MQ_weighted_)- Firstly, as the raw data of the subtest separated are represented by different units of measure, they were initially transformed (using Equation 2) as done previously in the sum of the standard scores. After this, the sums of the factor loadings of each subtest extracted of the Confirmatory Factor Analysis were calculated. Thirdly, item’s factor loadings were standardized by the sum of factor loadings. Then, the factor score was computed by the sum of each item score transformed (using Equation 2) by multiplying the standardized factor loading by the score of the item. For instance, in a KTK hypothetical case items factor (Factor loading – WB = 0.80; SJ = 0.90; MS = 0.40; HH = 0.60), the sum of the factor loading is 2.70 (Σ of the factor loading of all subtest). The standardized factors loading of the items are: WB– 0.80/2.70 = 0.30; SJ – 0.90/2.70 = 0.33; MS – 0.40/2.70 = 0.15; HH – 0.60/2.70 = 0.22. In the end, assuming this hypothetical example that subject one scored the highest score (in this case, it would have a transformed score = 1) in all subtests, the weighted factor score of the subject is 1 [(1*0.30)+(1*0.33)+(1*0.15)+(1*0.22)].

The refined method was computed by the factor extraction of the Confirmatory Factor Analysis. This procedure was computed using “predict() function” by the lavaan package (Rosseel, 2012). The main purpose of the “predict function” is to compute (or “predict”) estimated values for the latent variables (MQ_refined_) in the model (factor scores).

Since the units of measurement of the factor scores, extracted by the sum of the scores, sum of the standard scores, weighted method, and refined method values, are different, we used the Pearson’s correlation to verify the association between the methods. Moreover, the participants were grouped according to the levels of MC, as defined by their performance rates. The groups were formed by 0–20% (performance rates <20%), 20–40% (performance rates >20% and ≤40%), 40–60% (performance rates >40% and ≤60%), 60–80% (performance rates >60% and ≤80%), and 80–100% (performance rates >80%) using all methods. After that, a confusion matrix was generated using caret package to compare the classification of the standard, refined, and weighted methods with the sum method.

Two-way ANOVAs were used to compare sex and age differences in each subtest of the KTK. In addition, like other studies (e.g., Suppiah et al., 2016), partial eta-squared (ηp^2^) was used as a measure of effect size on the two-way ANOVA and classified using the following scale (small: 0.01; moderate: 0.09; large: 0.25). All analyses were conducted using α = 5%.

All the analyzes were performed in RStudio Version 1.1.463 for Windows that is an integrated development environment (IDE) for *R*.

## Results

### Descriptive Analysis

An overview of the raw scores of the subtests separated by age are presented in Tables 1–4.

**TABLE 1:** Descriptive analysis of the walking backwards subtest by age and sex.

**Age**	**All Sample**					**Male**					**Female**				
	***n***	**Max**	**Min**	**Mean**	***SD***	***n***	**Max**	**Min**	**Mean**	***SD***	***n***	**Max**	**Min**	**Mean**	***SD***
5 year-old	39	33	0	14.92	9.45	22	33	0	14.41	10.50	17	31	0	15.59	8.17
6 year-old	67	52	5	24.49	11.56	34	52	6	25.32	11.93	33	51	5	23.64	11.27
7 year-old	122	56	5	30.55	11.72	59	50	5	31.66	10.86	63	56	5	29.51	12.46
8 year-old	155	60	7	33.85	12.73	58	60	7	34.72	12.85	57	59	7	32.96	12.66
9 year-old	113	67	12	37.96	11.83	58	67	14	38.57	11.54	55	65	12	37.33	12.19
10 year-old	109	65	13	40.26	12.41	51	65	14	39.61	12.84	58	61	13	40.83	12.11
Total sample	565	67	0	32.78	13.74	282	67	0	33.04	13.74	283	65	0	32.52	13.77

**n*, sample size; Max, maximum value; Min, minimum value; *SD*, standard deviation.*

**TABLE 2:** Descriptive analysis of the jumping sideways subtest by age and sex.

**Age**	**All sample**					**Male**					**Female**				
	***n***	**Max**	**Min**	**Mean**	***SD***	***n***	**Max**	**Min**	**Mean**	***SD***	***n***	**Max**	**Min**	**Mean**	***SD***
5 year-old	39	46	12	21.79	8.52	22	46	12	20.41	9.21	17	35	14	23.59	7.53
6 year-old	67	48	7	27.24	8.55	34	48	10	28.76	9.01	33	41	7	25.67	7.88
7 year-old	122	68	6	35.90	12.84	59	68	15	36.76	11.52	63	65	6	35.10	14.01
8 year-old	155	68	5	38.57	13.56	58	68	9	40.79	14.70	57	64	5	36.30	12.00
9 year-old	113	73	8	44.12	13.58	58	72	15	43.31	12.14	55	73	8	44.96	15.01
10 year-old	109	73	22	50.25	10.68	51	73	29	50.53	11.31	58	68	22	50.00	10.18
Total sample	565	73	5	38.85	14.60	282	73	9	39.19	14.46	283	73	5	38.52	14.75

**n*, sample size; Max, maximum value; Min, minimum value; *SD*, standard deviation.*

**TABLE 3:** Descriptive analysis of the moving sideway subtest by age and sex.

**Age**	**All sample**					**Male**					**Female**				
	***n***	**Max**	**Min**	**Mean**	***SD***	***n***	**Max**	**Min**	**Mean**	***SD***	***n***	**Max**	**Min**	**Mean**	***SD***
5 year-old	39	35	9	20.97	6.09	22	32	12	20.64	5.42	17	35	9	21.41	7.02
6 year-old	67	45	11	28.07	7.59	34	41	12	29.15	7.64	33	45	11	26.97	7.50
7 year-old	122	53	17	32.97	7.95	59	53	20	33.27	8.06	63	53	17	32.68	7.91
8 year-old	155	58	15	34.21	8.55	58	55	16	34.74	9.09	57	58	15	33.67	8.02
9 year-old	113	61	12	36.86	9.92	58	53	19	35.71	8.17	55	61	12	38.07	11.44
10 year-old	109	68	23	43.68	9.50	51	68	25	43.92	9.34	58	67	23	43.47	9.72
Total sample	565	68	9	34.66	10.48	282	68	12	34.52	10.17	283	67	9	34.80	10.80

**n*, sample size; Max, maximum value; Min, minimum value; *SD*, standard deviation.*

**TABLE 4:** Descriptive analysis of the hopping for height subtest by age and sex.

**Age**	**All sample**					**Male**					**Female**				
	***n***	**Max**	**Min**	**Mean**	***SD***	***n***	**Max**	**Min**	**Mean**	***SD***	***n***	**Max**	**Min**	**Mean**	***SD***
5 year-old	39	41	6	20.77	9.78	22	41	6	20.86	9.70	17	39	6	20.65	10.18
6 year-old	67	55	11	31.69	9.91	34	55	14	34.62	9.39	33	47	11	28.67	9.65
7 year-old	122	63	11	37.49	19.48	59	63	13	40.95	10.64	63	56	11	34.25	9.29
8 year-old	155	68	17	40.77	12.35	58	68	18	43.19	11.37	57	66	17	38.30	12.92
9 year-old	113	77	23	46.73	12.90	58	77	23	51.24	13.79	55	74	25	41.96	9.98
10 year-old	109	78	17	52.22	15.32	51	78	17	54.76	16.71	58	77	26	49.98	13.75
Total sample	565	78	6	41.00	14.92	282	78	6	43.70	15.54	283	77	6	38.32	13.78

**n*, sample size; Max, maximum value; Min, minimum value; *SD*, standard deviation.*

### Correlation Between Subtests

**FIGURE 1:**
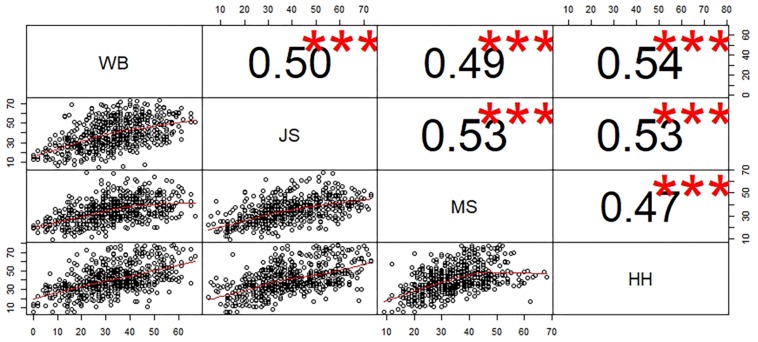
Scatter Plot and correlation coefficient of the subtests of the KTK. ^∗∗∗^*p* < 0.001.

The correlations between all subtests of the KTK (Figure 1) were positive, weak to moderate (from 0.47 to 0.54), and significant (*p* < 0.0001).

### Construct Validity, Reliability, and Invariance Analysis

**FIGURE 2:**
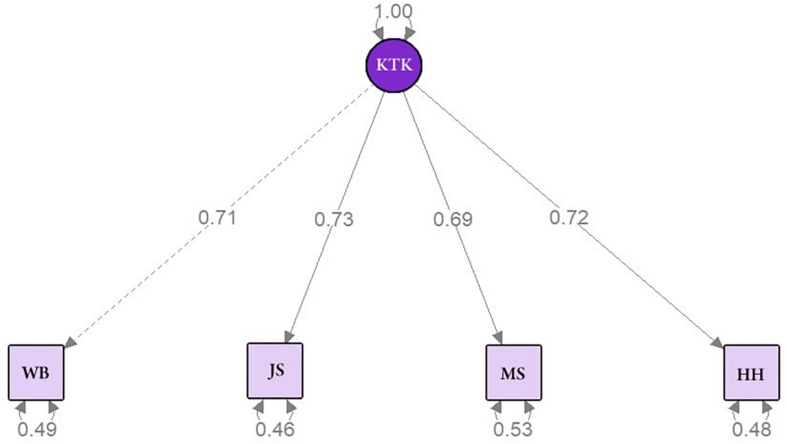
Adjustment indices for the CFA and factor loads for the total sample.

Figure 2 shows the CFA path model of all sample results that confirmed the existence of a single latent factor (MC) for the KTK. The results of the measures for the analysis were considered adequate (χ^2^ = 5.086, *p* = 0.079, CFI = 0.995, TLI = 0.986, RMSEA = 0.052, SRMR = 0.015).

**FIGURE 3:**
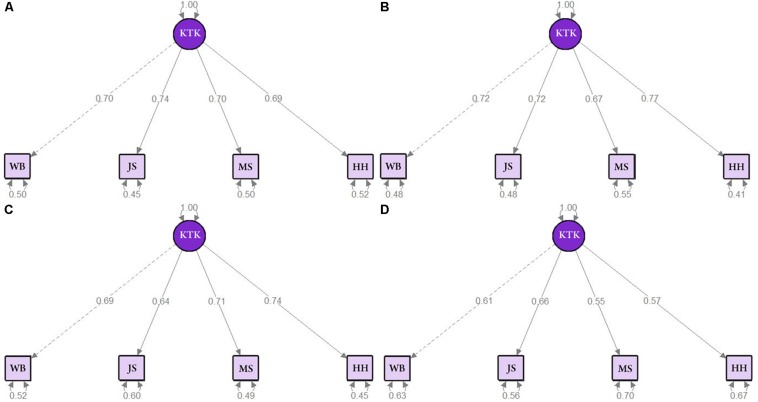
Adjustment indices for the CFA and factor loads for **(A)** male sample, **(B)** female sample, **(C)** the 5 to 7 age group, and **(D)** the 8 to 10 age group.

In addition, Figures 3A,B show the CFA path model for male and female sample, respectively. In summary, the results confirmed the existence of a single latent factor (MQ) for the KTK in both sexes. Moreover, the results of the measures for the analysis were considered adequate for male (χ^2^ = 2.733, *p* = 0.255, CFI = 0.998, TLI = 0.993, RMSEA = 0.036, SRMR = 0.016) and female (χ^2^ = 3.255, *p* = 0.196, CFI = 0.997, TLI = 0.990, RMSEA = 0.047, SRMR = 0.016).

Figures 3C,D show the CFA path model for 5–7 years old group and 8–10 years old group sample, respectively. In summary, the results confirmed the existence of a single latent factor (MQ) for the KTK in both age groups. Moreover, the results of the measures for the analysis were considered adequate for 5 to 7 years old group (χ^2^ = 0.340, *p* = 0.844, CFI = 1.000, TLI = 1.020, RMSEA = 0.000, SRMR = 0.006) and 8 to 10 years old group (χ^2^ = 5.881, *p* = 0.053, CFI = 0.981, TLI = 0.943, RMSEA = 0.076, SRMR = 0.027).

The Cronbach’s alpha was conducted for all the subtests of the KTK in which the value was 0.80 for all sample, 0.79 for male, and 0.81 for female.

In the model testing metric invariance (Tables 5, 6), Configural invariance showed that the number of latent variables and the pattern of loadings of latent variables on indicators is similar across the sexes and age groups. Weak invariance (also known as metric invariance) indicated that the magnitude of the loadings is similar across age groups, but not across sexes. Moreover, strong invariance (also known as scalar invariance) showed that item intercepts are statistically different across the sexes and age groups. In the end, strict invariance showed that residual variances are not similar across sexes and age groups.

**TABLE 5:** Results of measurement invariance across sex.

**Models**	**χ^2^**	**Δχ^2^**	**df**	**Δ df**	**RMSEA (90% CIs)**	**Δ RMSEA**	**CFI**	**Δ CFI**	**Comparisons**
Single-group solutions									
Male	2.733		6		0.036 (0.000, 0.129)		0.998		
Female	3.255		6		0.047 (0.000, 0.136)		0.997		
Model 1 configural invariance	5.988		4		0.042 (0.000, 0.106)		0.997		
Model 2 metric invariance	6.110	0.1216	7	3	0.000 (0.000, 0.067)	−0.042	1.00	0.003	Model 2 vs. Model 1
Model 3 scalar invariance	32.550	26.440^∗∗^	10	3	0.089 (0.056, 0.124)	0.089	0.968	−0.032	Model 3 vs. Model 2
Model 4 strict invariance	44.662	12.112^∗^	14	4	0.088 (0.060, 0.118)	−0.001	0.956	−0.012	Model 4 vs. Model 3

*χ^2^, chi-square goodness of fit; df, degrees of freedom; RMSEA, root mean square error of approximation; 90% CIs, 90% confidence intervals for RMSEA; CFI, comparative fit index; Δχ^2^, chi-square goodness of fit difference; Δ df, degrees of freedom difference; ΔCFI, CFI difference; ΔRMSEA, RMSEA difference. ^∗^*p* < 0.01 and ^∗∗^*p* < 0.001.*

**TABLE 6:** Results of measurement invariance across age groups.

**Models**	**χ^2^**	**Δχ^2^**	**df**	**Δ df**	**RMSEA (90% CIs)**	**Δ RMSEA**	**CFI**	**Δ CFI**	**Comparison**
**Single-group solutions**									
5 to 7 years old	0.340		2		0.000 (0.000, 0.074)		1.000		
8 to 10 years old	5.881		2		0.076 (0.000, 0.150)		0.981		
Model 1 configural invariance	6.220		4		0.044 (0.000, 0.108)		0.995		
Model 2 metric invariance	8.683	2.463	7	3	0.029 (0.000, 0.082)	0.015	0.996	−0.001	Model 2 vs. Model 1
Model 3 scalar invariance	10.611	1.928	10	3	0.015 (0.000, 0.067)	0.014	0.999	−0.003	Model 3 vs. Model 2
Model 4 strict invariance	57.735	47.124^∗∗^	14	4	0.105 (0.078, 0.134)	−0.090	0.903	0.096	Model 4 vs. Model 3

*χ^2^, chi-square goodness of fit; df, degrees of freedom; RMSEA, root mean square error of approximation; 90% CIs, 90% confidence intervals for RMSEA; CFI, comparative fit index; Δχ^2^, chi-square goodness of fit difference; Δ df, degrees of freedom difference; ΔCFI, CFI difference; ΔRMSEA, RMSEA difference. ^∗^*p* < 0.01 and ^∗∗^*p* < 0.001.*

### Analysis of the Factor Scores

**FIGURE 4:**
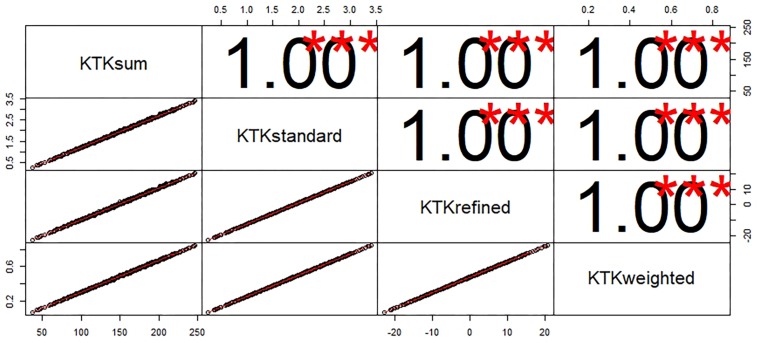
Scatter Plot, histogram and correlation coefficient of the factor score methods. ^∗∗∗^*p* < 0.001.

The analysis of the correlation between factor score methods showed that all methods were statistically significant, positive, and large (Figure 4).

**FIGURE 5:**
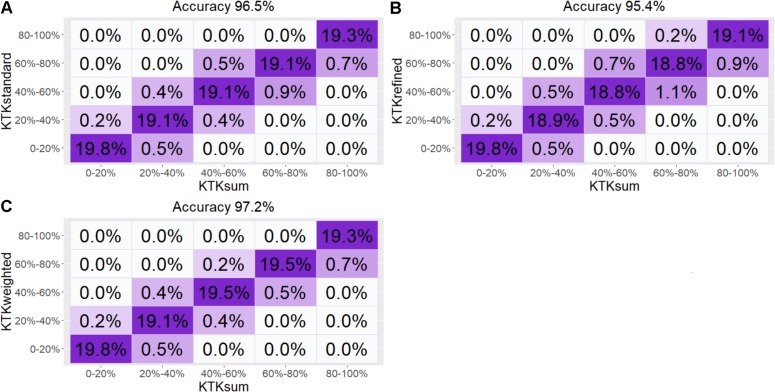
The confusion matrix generated to compare the classification generated by methods. **(A)** Sum of raw scores with standard methods. **(B)** Sum of raw scores with refined methods. **(C)** Sum of raw scores with Weighted methods.

The confusion matrix generated to compare the classification generated by performance rates of the standard, refined, and weighted methods with the sum of raw scores method showed that the standard, refined, and weighted methods present classification practically similar to the sum method with an accuracy in the classification of 95.4% to 97.2% (Figure 5).

### Sex and Ages Analysis

**FIGURE 6:**
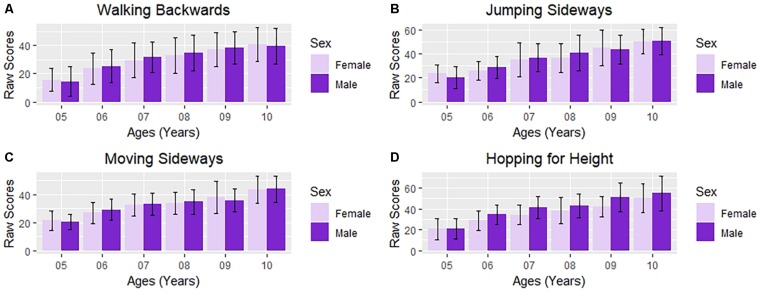
Bar Plot of the performance in each subtest of the KTK by age and sex. **(A)** Walking backwards; **(B)** jumping sideways; **(C)** moving sideways; **(D)** hopping for height.

The two-way ANOVA (2 sex and 5 age groups) analysis of the Walking Backward (Figure 6A) indicated main effects of Age [*F*(5,557) = 37.738; *p* < 0.01; η^2^ = 0.253] with large effect size, in which Tukey *post hoc* test showed that the performance of older children was significantly better than younger children except for 7–8 year-old (*p* = 0.274), 8 to 9 year-old (*p* = 0.099), and 9 to 10 year-old (*p* = 0.709). The sex [*F*(1,557) = 0.908; *p* = 0.341; η^2^ = 0.002] and interaction [*F*(1,557) = 0.263; *p* = 0.608; η^2^ < 0.001] analyses were no significant.

The two-way ANOVA (2 sex and 5 age groups) analysis of the Jumping Sideways (Figure 6B) indicated main effects of Age [*F*(5,557) = 53.115; *p* < 0.01; η^2^ = 0.323], with large effect size, in which Tukey *post hoc* test showed that the performance of older children was significantly better than younger children except for 5 to 6 year-old (*p* = 0.222) and 7 to 8 year-old (*p* = 0.534). The sex [*F*(1,557) = 1.391; *p* = 0.239; η^2^ = 0.002] and interaction [*F*(1,557) = 0.101; *p* = 0.750; η^2^ < 0.001] analyses were not significant.

The two-way ANOVA (2 sex and 5 age groups) analysis of the Moving Sideways (Figure 6C) indicated main effects of Age [*F*(5,557) = 53.095; *p* < 0.01; η^2^ = 0.323], with large effect size, in which Tukey *post hoc* test showed that the performance of older children was significantly better than younger children except for 7 to 8 year-old (*p* = 0.881) and 8 to 9 year-old (*p* = 0.193). The sex [*F*(1,557) = 0.052; *p* = 0.820; η^2^ < 0.001] and interaction [*F*(1,557) = 0.492; *p* = 0.483; η^2^ = 0.001] analyses were not significant.

The two-way ANOVA (2 sex and 5 age groups) analysis of the Hopping for Height (Figure 6D) indicated the main effects of Ages [*F*(5,557) = 56.880; *p* < 0.01; η^2^ = 0.338], with large effect size, and sex [*F*(1,557) = 34.817; *p* < 0.01; η^2^ = 0.059], with small to moderate effect size, in which Tukey *post hoc* test showed that the performance of older children was significantly better than younger children, except for 7 to 8 year-old (*p* = 0.286). In addition, boys showed better performance than girls (p < 0.001). The interaction [*F*(1,557) = 0.492, *p* = 0.483, η^2^ = 0.001] analyses was not significant.

### Interpretative Test Parameters

Based on the MQ calculated by sum of the raw scores of the subtest, the interpretative parameters are shown in Table 7.

**TABLE 7:** Interpretative parameters of the motor quotient using sum of raw scores subtests by age and sex.

**Age**	**All sample**					**Male**					**Female**				
	***n***	**Max**	**Min**	**Mean**	***SD***	***n***	**Max**	**Min**	**Mean**	***SD***	***n***	**Max**	**Min**	**Mean**	***SD***
5 year-old	39	136	37	78.46	25.73	22	136	46	76.32	26.33	17	126	37	81.24	25.46
6 year-old	67	183	45	111.49	26.85	34	183	62	117.85	28.09	33	147	45	104.94	24.21
7 year-old	122	221	68	136.91	30.12	59	221	68	142.64	28.51	63	195	75	131.54	30.82
8 year-old	155	217	65	147.39	33.41	58	209	65	153.45	32.49	57	217	76	141.23	33.47
9 year-old	113	228	69	165.66	31.88	58	228	93	168.83	31.81	55	221	69	162.33	31.91
10 year-old	109	247	87	186.40	33.75	51	246	119	188.82	33.27	58	247	87	184.28	34.31
Total sample	565	247	37	147.29	42.84	282	246	46	150.44	42.89	283	247	37	144.16	42.65

**n*, sample size; Max, maximum value; Min, minimum value, *SD*, standard deviation.*

## Discussion

The present study aimed to investigate the factor structure of KTK for a Brazilian sample; and, compared four possibilities of calculating the factorial score of the test, precisely the sum of the scores, sum of the standard scores, weighted method, and the refined method.

In summary, the results show that the psychometric properties of KTK from a sample composed of Brazilian children were well adjusted for the total sample and separated by sex and age groups. Using the CFA was possible, identifying in the instrument a single latent factor, which can be named motor quotient to assess children and adolescents MC. However, the results do not confirm the invariance between the sexes. In addition, an interesting result found by us was that the different methods of calculating factor scores present a high correlation with each other. Thus, our result indicating that the sum of the raw scores of the subtests, which is the simplest way of calculating the factor score of the KTK, can be used.

The factorial structure of the KTK was shown to be well adjusted to the model for all sample and separated by sex, which suggests that the instrument has appropriate capacity to assess the MC of the group from this population aged between 5 and 10. The present study advances in the current knowledge by providing the first psychometric analysis of the KTK for Brazilian children. As pointed out by a previous study (Ribeiro et al., 2012), there was a emergent need for the validation since the test has been used for research and clinical purposes in Brazil. Internationally, the study of Rudd et al. (2016) with Australian children, also used factorial analysis to investigate the factorial structure of KTK, found a model that adequately fits the data, in which the four tasks had a strong effect on the latent MC variable. The results obtained by these authors give support to the findings of the present study for the Brazilian sample and confirm KTK as an adequate tool to measure MC in different population contexts. Although it is necessary to consider the specificities of each population, which undergo different environmental, social, and cultural influences (Robinson et al., 2015), the KTK seems to be capable of measuring levels of MC even in different contexts, despite the results pointing to variations in performance for samples from different countries (Graf et al., 2005). The validity of its construct, combined with its practicality, makes the KTK a viable instrument to be used in different contexts (research and, professional practice, among others), as it is a simple and objective test, with low operational cost and with low interference on physical fitness.

We conducted multiple groups confirmatory factor analysis to investigate the degree to which measures are invariant across sex and age groups, and our results showed that the assumption of invariance across sex and age groups were not confirmed. As well documented, the measurement of invariance has a very important implication for the interpretation of differences between groups (e.g., sex and age groups in our study). In this sense, as the invariance assumptions have not been confirmed, we cannot assume a stable relationship between the construct and the test score. Thus, the observed mean differences between sex and age in KTK may be either due to differences in underlying constructs or due to the different relations between latent constructs and scores (Hirschfeld and von Brachel, 2014). Therefore, our results have shown that, as invariance was not found for sex and age, further investigations using KTK need to be adjusted by sex and age. One way to make this adjustment would be the use of normative tables, as presented by us in the present study (e.g., Table 7).

Two important limitations are found to compute the factor score in the KTK: (1) The subtests have different measuring units; (2) The weight is given to each subtest in the factor score calculations. Thus, methods that control such limitations may be of great value to the quality of the measure. On the other hand, more robust methods that control these disadvantages, usually have limitations, such as not being simple enough for their wide practical use. Our result showed that the correlation between the different methods is statistically significant, positive, and strong. In addition, the confusion matrix generated showed that the three methods (standard, refined, and weighted methods) used to calculate the KTK factor score present classification practically similar to the sum of raw scores of the subtest’s methods with an accuracy of 95.4% to 97.2%. Thus, it is possible to assume that the sum of raw scores of the subtests of the KTK, which is a simple and easy method, can be used as a measure of KTK factor score.

Regarding the possible limitations presented by the present study, it is important that the results obtained are not seen as ultimate due to some factors. The first one concerns the sample, which has regional characteristics. However, it is worth mentioning that, to the best of our knowledge, this is the first study that has sought to investigate the validity of the test in Brazilian children and adolescents. Another limiting aspect is the fact that KTK only tests MC in children, with few locomotion actions and almost no manipulation of objects. Considering the influence of general motor competence in the adoption of a more active lifestyle by children, it might be interesting to use a complementary test, such as TGMD-2 (Ulrich, 2000). Another limitation concerns the fact that no information on whether the children practiced sports or had any level of physical activity was considered. Children with more time dedicated to motor practices could have taken a certain advantage in the test, influencing the task scores (Vandorpe et al., 2012). In future studies, objective methods for assessing the level of physical activity should be employed. The influence of the children’s BMI on scores also needs to be better investigated, since a previous study (D’Hondt et al., 2011) found that overweight in childhood influences KTK performance negatively. In the end, the fact that KTK has a good factorial structure does not indicate that conceptually implies that it assesses the motor competence thoroughly. For this reason, there is a suggestion that KTK can be combined with TGMD-2 (Rudd et al., 2016) to theoretically make the motor competence construct more robust and therefore, a better motor competence assessment.

In summary, the results of the present study extend the current knowledge regarding the use of KTK as a tool to measure MC in children and adolescents, especially concerning the Brazilian reality. Our result showed that the sum of raw scores method has a statistic, positive, and large correlation with other robust methods to calculate the factor score, and can therefore be interpreted as a simple and adequate method for the interpretations of KTK results.

## Conclusion

The results obtained in the present study establish parameters to extend the use of the test in Brazil and may contribute in a way that new research could be conducted to establish validity across the entire country and yet normative values for the Brazilian population. Normative values are necessary to extend the use to KTK in Brazil; reference scores should be produced according to geographic, cultural and social realities. However, in addition to the applications in the science field, it is essential that such information reaches the knowledge of physical education teachers and sports coaches, in order to give them conditions to apply the tests in the field, using the obtained results to support the elaboration and the execution of programs aiming to develop motor skills in children and adolescents.

## Data Availability Statement

The datasets generated for this study are available on request to the corresponding author.

## Ethics Statement

The studies involving human participants were reviewed and approved by the Universidade Federal de Viçosa (26874614.1.0000.5153). Written informed consent to participate in this study was provided by the participants’ legal guardian/next of kin.

## Author Contributions

JM, ML, and MJ collected the data. NV and GL participated with MA on the conception and design of the study. MA analyzed the data. All authors participated in the interpretation of the results, drafted and revised the manuscript, and approved the final revision of the manuscript.

## Conflict of Interest

The authors declare that the research was conducted in the absence of any commercial or financial relationships that could be construed as a potential conflict of interest.
